# Integrative bioinformatics analysis and experimental validation of key biomarkers for risk stratification in primary biliary cholangitis

**DOI:** 10.1186/s13075-023-03163-y

**Published:** 2023-10-02

**Authors:** Siyuan Tian, Yinan Hu, Miao Zhang, Kemei Wang, Guanya Guo, Bo Li, Yulong Shang, Ying Han

**Affiliations:** State Key Laboratory of Cancer Biology, National Clinical Research Center for Digestive Diseases, Xijing Hospital of Digestive Diseases, Air Force Medical University, Xi’an, 710032 Shaanxi China

**Keywords:** Primary biliary cholangitis, GEO database, Biomarker, Bioinformatics analysis, Risk stratification

## Abstract

**Background:**

Primary biliary cholangitis (PBC) is an autoimmune liver disease, whose etiology is yet to be fully elucidated. Currently, ursodeoxycholic acid (UDCA) is the only first-line drug. However, 40% of PBC patients respond poorly to it and carry a potential risk of disease progression. So, in this study, we aimed to explore new biomarkers for risk stratification in PBC patients to enhance treatment.

**Methods:**

We first downloaded the clinical characteristics and microarray datasets of PBC patients from the Gene Expression Omnibus (GEO) database. Differentially expressed genes (DEGs) were identified and subjected to enrichment analysis. Hub genes were further validated in multiple public datasets and PBC mouse model. Furthermore, we also verified the expression of the hub genes and developed a predictive model in our clinical specimens.

**Results:**

A total of 166 DEGs were identified in the GSE79850 dataset, including 95 upregulated and 71 downregulated genes. Enrichment analysis indicated that DEGs were significantly enriched in inflammatory or immune-related process. Among these DEGs, 15 risk-related genes were recognized and further validated in the GSE119600 cohort. Then, TXNIP, CD44, ENTPD1, and PDGFRB were identified as candidate hub genes. Finally, we proceeded to the next screening with these four genes in our serum samples and developed a three-gene panel. The gene panel could effectively identify those patients at risk of disease progression, yielding an AUC of 0.777 (95% CI, 0.657–0.870).

**Conclusions:**

In summary, combining bioinformatics analysis and experiment validation, we identified TXNIP, CD44, and ENTPD1 as promising biomarkers for risk stratification in PBC patients.

**Supplementary Information:**

The online version contains supplementary material available at 10.1186/s13075-023-03163-y.

## Introduction

Primary biliary cholangitis (PBC) is an autoimmune liver disease, characterized by female predominance, non-suppurative destruction of small bile ducts, and specific anti-mitochondrial antibodies (AMAs) [1, 2]. The etiology and pathogenesis of PBC are not well understood and may be associated with immune dysregulation, environment factors, and genetic susceptibility [3]. In recent years, the incidence and prevalence of PBC are increasing worldwide. A recent meta-analysis showed that the annual incidence of PBC varied from 0.23 to 5.31 per 100,000, with a prevalence ranging from 1.91 to 4.02 per 100,000 [4]. Moreover, as the disease progresses, it can eventually develop into cirrhosis and liver failure [5]. Of note, there are data suggesting the median survival of symptomatic patients was only 7.5 years [6].

Currently, ursodeoxycholic acid (UDCA) is the only first-line treatment for this disease, which could improve biochemical indexes and prolong transplant-free survival of patients [7]. However, yet up to 40% of patients with PBC have an incomplete response to UDCA [8]. These patients are identified as high-risk patients, who will go on to develop into cirrhosis and eventually progress to death due to the complications [9]. Recent guidelines recommend obeticholic acid (OCA) for use in combination with UDCA in PBC patients with inadequate response to UDCA [10]. A randomized placebo-controlled trial also showed the benefits of UDCA in combination with bezafibrate [11]. Nevertheless, it conventionally takes 12 months to determine the UDCA response of patients [12–14]. In such cases, effective treatments are lacking for those high-risk patients. In fact, it is high-risk patients that need early interventions like a combination of medications. Thus, reliable markers that can early identify high-risk patients are urgently needed for refining therapeutic strategies.

Previous studies have shown that some baseline characteristics could be used to predict the UDCA response, such as age, sex, autoantibodies, and biochemical indicators [15–18]. In our previous studies, we also attempted to integrate relevant pretreatment clinical parameters to predict the inadequate UDCA response for PBC patients [19]. Although these features are frequently used as indicators of disease progression, they have fallen short of depicting the true nature of the illness. At present, high-throughput methods, like genomics and transcriptomics, have been widely utilized to identify key genes or pathways to elucidate the molecular mechanisms of multiple diseases [20–23]. The resulting omics information might offer access to estimate the risk of disease progression or clinical outcomes. Over the years, several non-coding RNAs were confirmed to be involved in pathogenesis and might become potential therapeutic target for PBC patients. For example, miR-506 may impair bile secretion in PBC by inhibiting Cl^−^/HCO_3_^−^ anion exchanger 2 (AE2) expressions [24]. miR-425 was identified a key regulator of inflammatory cytokine production in CD4^+^ T cells [25]. Our previous studies also demonstrated that a decline in LAMP-2 predicted the UDCA response of PBC patients. However, these individual markers remain to be verified in a large cohort of patients with PBC. With the aid of bioinformatics datasets of PBC deposited in GEO platform, we may achieve an in-depth mining of key genes or pathways which are closely related to the development of PBC.

Therefore, this study was carried out to explore novel predictors of risk stratification for PBC patients. We firstly downloaded GSE79850 [26] and GSE119600 [27] from the GEO database. R language was utilized to standardize and analyze the microarray datasets to obtain risk-related genes. These genes were also examined in the PBC mouse model. Next, we validated these key biomarkers using clinical serum samples in our center and further explored the predictive value in risk stratification of PBC.

## Methods

### Data collection

Series matrix files and corresponding clinical information of two datasets (GSE79850 and GSE119600) were downloaded from the GEO database (https://www.ncbi.nlm.nih.gov/geo/). GSE79850 was based on the GPL 19965 annotation platform, which included 16 PBC liver tissue samples and 8 control samples. These PBC patients were divided into high-risk or low-risk groups using clinical outcomes after long-term follow-up. High-risk patients were defined as non-responders to treatment with UDCA at 1 year using Paris-I criteria [14] and subsequently requiring liver transplantation. Low-risk patients were defined as responders to UDCA at 1 year and still responsive after a minimum of 15 years of follow-up. The GSE79850 dataset was firstly used to explore biomarkers for the disease progression. Next, the GSE119600 dataset including 137 blood samples (90 PBC samples and 47 healthy controls) was employed as a validation analysis dataset.

### Identification of differentially expressed genes (DEGs)

The original files were firstly normalized by using Robust Multiarray Average (RMA) algorithm. Next, the “limma” package of R software (version. 4.0.1) was used to conduct differential analysis. The significance threshold was set at |Log2FC|> 1 and adjusted *P*-value < 0.05. To visualize the identified DEGs, R software was used to make heatmaps and volcano plots by using the “pheatmap” and “ggplot2” packages.

### Functional enrichment analysis

To further explore the potential biological functions of DEGs, we performed the functional enrichment analysis. The “clusterProfler” package was employed to conduct Gene Ontology (GO) and Kyoto Encyclopedia of Genes and Genomes (KEGG) pathway enrichment analysis.

### Experimental animals

Our dnTGF-βRII mice were a generous gift from Pro. ZX Lian (Guangdong Provincial People’s Hospital). dnTGF-βRII mice were bred onto a C57BL/6 (B6) strain background at the animal facilities of the Air Force Medical University [28, 29]. Male heterozygous dnTGFβ-RII mice were bred with female B6 mice to obtain female heterozygous dnTGFβ-RII mice, which were genotyped to confirm the dnTGFβ-RII gene in their genomic DNA by the detection of the CD4 promoter at the age of 3–4 weeks [29]. All mice were maintained in individually ventilated cages under specific pathogen-free conditions. They were fed a standard chow diet ad libitum with free access to water. At 12–14 weeks of age, animals were sacrificed by anesthesia with CO_2_ and their livers were processed as experimental requirements. The animal study protocol was approved by the Animal Welfare and Ethics Committee of the Air Force Medical University (20230945).

### Histopathology

For hematoxylin and eosin staining, the whole liver tissue was firstly fixed with 4% paraformaldehyde. Then, fixed tissues were embedded in paraffin, sliced into 4-μm sections, and subjected to hematoxylin and eosin staining. The slides were scanned and digitalized using CaseViewer software (3DHISTECH, Budapest, Hungary).

### Clinical cohort for biomarker validation

Patients who were diagnosed as PBC from January 2021 to December 2021 were enrolled as another validation cohort. The diagnosis of PBC was based on the 2018 American Association for the Study of Liver Diseases PBC guidelines [6]. In the current study, patients were excluded if they had viral hepatitis (hepatitis B or C), steatohepatitis, or alcoholic liver disease. Patients complicated with primary sclerosing cholangitis, autoimmune hepatitis, and other autoimmune diseases were also excluded. Blood samples from each participant were collected at their first visit to outpatient clinic and centrifuged at 3000 rpm for 10 min at 25 °C. The upper serum layer was extracted and stored in − 80 °C for subsequent experiments. The clinical characteristics and biochemical indices were extracted from the electronic medical records. The detailed clinical information of the included patients is shown in Table 1. For PBC, the serum alkaline phosphatase and total bile acid are important indicators for judging the prognosis of patients, which can be used as a surrogate endpoint in clinical trials [30]. In the present study, we used the POISE criteria (ALP < 1.67 × upper limit of normal (ULN) and bilirubin < 1 × ULN) to distinguish high- or low-risk patients [31]. Informed consent written was obtained from all participants in this study and the study protocol was approved by the Ethics Committee of Xijing Hospital.

**Table 1:** The clinical information of the included patients with primary biliary cholangitis

**Characteristics**	**Low risk**	**High risk**	*P***-value**
	**(*N* = 42)**	**(*N* = 24)**	
Age (year)	53.31 ± 8.39	55.83 ± 8.83	0.253
Sex			0.101
Male	3 (7.1%)	5 (20.8%)	
Female	39 (92.9%)	19 (79.2%)	
RBC (× 10^9^/L)	4.46 (4.11, 4.59)	4.14 (3.75, 4.48)	0.021
HGB (g/L)	128.5 (121.8, 138.3)	129.5 (109.3, 135.5)	0.268
PLT (× 10^9^/L)	168.5 (131.3, 226.0)	120.5 (90.5, 173.3)	0.066
ALT (IU/L)	19.5 (12.8, 27.5)	39.5 (25.5, 83.5)	< 0.001
AST (IU/L)	30.0 (23.0, 36.5)	51.0 (37.0, 83.3)	< 0.001
ALB (g/L)	45.4 (42.8, 47.1)	42.6 (37.5, 45.8)	0.03
ALP (IU/L)	111.5 (87.5, 138.5)	214.0 (131.0, 299.3)	< 0.001
GGT (IU/L)	44.0 (24.8, 77.5)	129.5 (38.3, 320.5)	0.002
TBiL (μmol/L)	12.8 (9.6, 18.2)	27.7 (21.4, 42.7)	< 0.001

*RBC* red blood cell, *HGB* hemoglobin, *PLT* platelet, *ALT* alanine aminotransferase, *AST* aspartate aminotransferase, *ALB* albumin, *ALP* alkaline phosphatase, *GGT* gamma glutamyl transferase, *TBiL* total bilirubin

### RNA extraction and gene expression analysis

Total RNA was extracted from 200 μl of serum using Trizol reagent (Sigma, USA), and then, RNA was reverse transcribed into cDNA with a high-capacity cDNA reverse transcription kit (Takara). Quantitative real-time PCR (qRT-PCR) analyses were performed by SYBR Green premix pro Taq HS qRT-PCR kit (Accurate Biotechnology (Hunan) Co., Ltd) to validate gene expression, and the level of β-Actin served as an internal control. The relative expression of the target gene was calculated and normalized to the expression of the reference gene β-Actin. The primers’ sequences for qRT-PCR are shown in Supplementary Table 1.

### Statistical analysis

Data were analyzed by using R software (version. 4.0.1) and SPSS (version. 23.0). Continuous data was expressed as mean ± standard deviation or median and interquartile range. The Student *t* test or Mann–Whitney *U* test was used for analysis as appropriate. Categorical data was described as frequency (percentage) and chi-square or Fisher’s exact tests were used to analyze differences between groups. Bivariate analyses were performed using the Spearman correlation test. A logistic regression algorithm was used to construct the predictive model. The receiver operation characteristic (ROC) curve and calibration curve were employed to examine the discrimination and calibration of the model. ROC curves were generated by MedCalc software (version. 19.2.1), while calibration curves were drawn with the R package “rms”. Two-tailed *p*-value < 0.05 was deemed statistically significant.

## Results

### Identification of DEGs between healthy controls and PBC patients

**Fig. 1:**
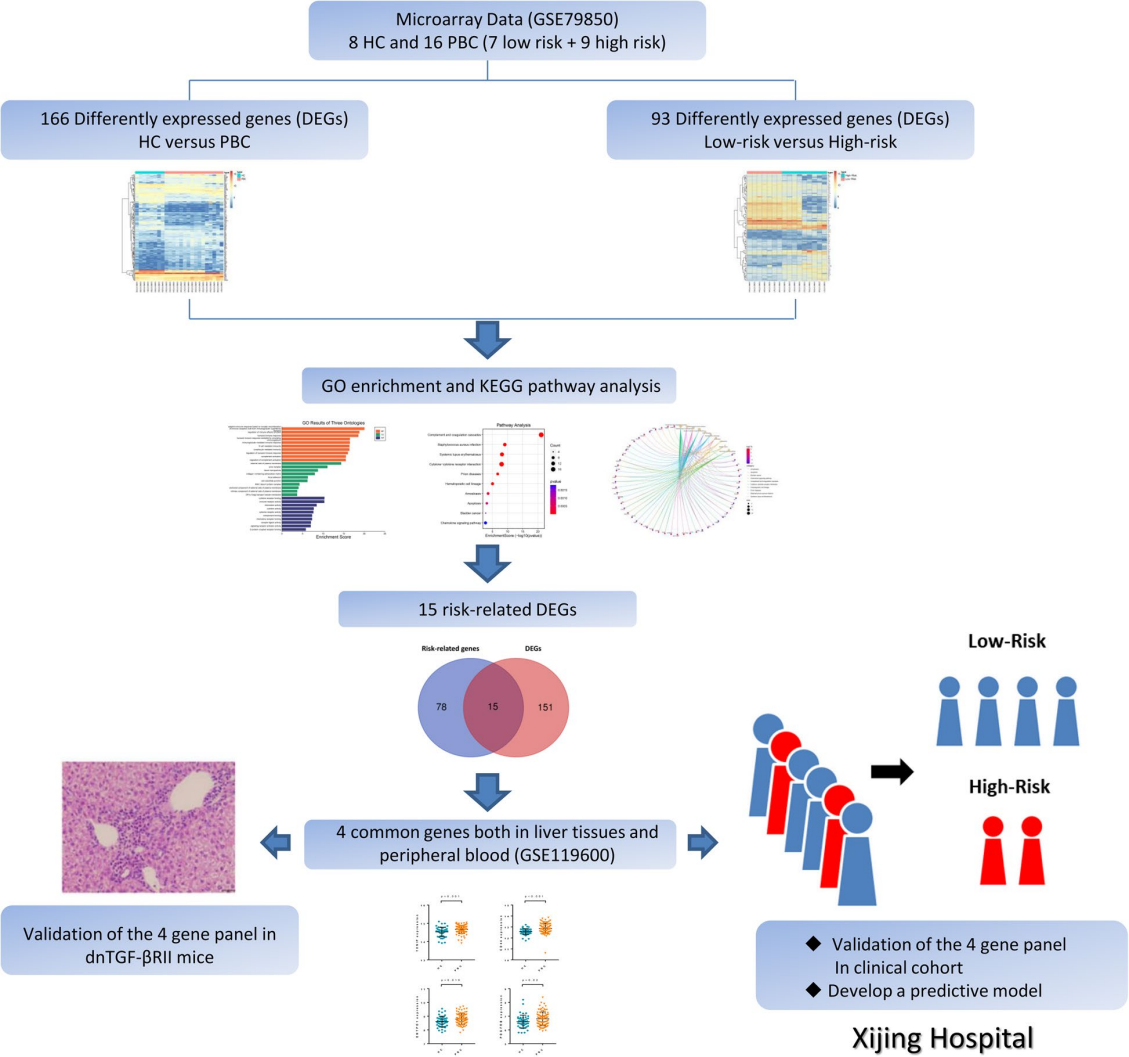
The workflow of this study

**Fig. 2:**
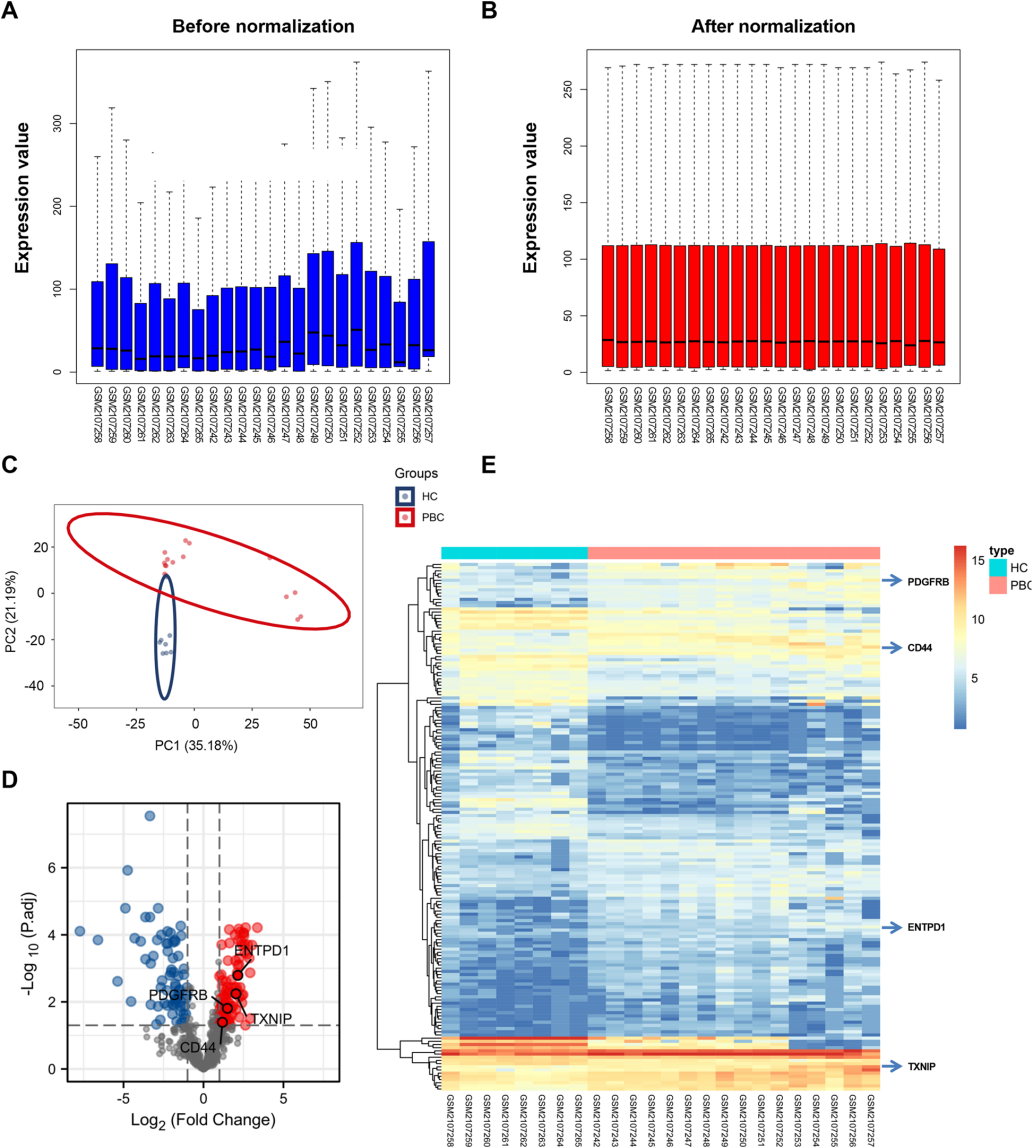
Normalization of the microarray datasets (GSE79850) and differential gene expression analysis. The gene expression value data before and after normalization (**A**, **B**). **C** Principal component analysis (PCA) of the samples. Blue dots represented the PCA values of 8 healthy controls, and red blots represented 16 PBC patients. **D** Volcano plots of differentially expressed genes. Blue circles represented downregulated genes and red circles represented upregulated genes. The screening threshold was set as |Log2FC|> 1 and adjusted *P*-value < 0.05 (TXNIP: |Log2FC|= 2.033 *P*-adj = 0.006, ENTPD1: |Log2FC|= 2.149 *P*-adj = 0.002, CD44: |Log2FC|= 1.183 *P*-adj = 0.041, PDGFRB: |Log2FC|= 1.500 *P*-adj = 0.016). **E** Heatmap analysis of all differentially expressed genes

Figure 1 shows the overall workflow of this study and the detailed information of included clinical or animal specimens is summarized in Table 2. Firstly, the gene expression series GSE79850 was normalized, and the results are shown in Fig. 2A, B. Principal component analysis results showed a good separation between PBC samples and Healthy controls (Fig. 2C). Then, we used the “limma” package to calculate the differential genes between the two groups (adjusted *P*-value < 0.05 and |Log2FC|> 1). Compared with HC controls, a total of 166 DEGs were considered to be differentially expressed in PBC samples, including 95 upregulated genes and 71 downregulated genes (Fig. 2D). The heatmap showed distinct gene expression patterns between two groups (Fig. 2E).

**Table 2:** Information of included clinical and animal specimens

Data source	Sample type	Sample size
GSE79850	Liver tissue	8 HC + 16 PBC patients
GSE119600	Peripheral blood	47 HC + 90 PBC patients
Animal model	Liver tissue and peripheral blood	6 WT + 6 dnTGF-βRII mice
Clinical samples	Peripheral blood	66 PBC patients

### Functional enrichment analysis

**Fig. 3:**
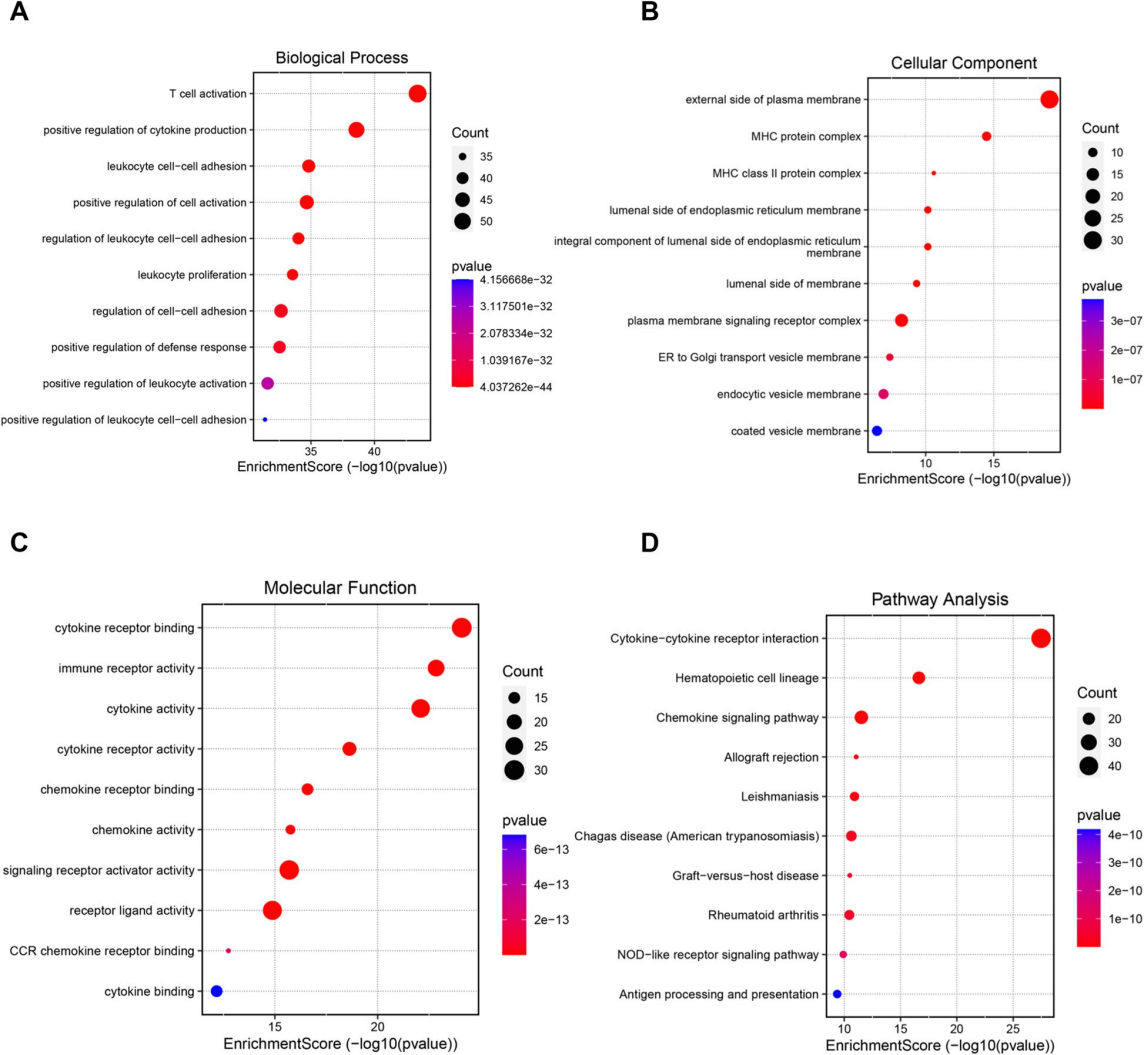
Functional enrichment analysis of differentially expressed genes. **A**–**C** GO enrichment analysis contained three categories: biological process, molecular function, and cellular component. **D** KEGG pathway enrichment analysis. The top 10 functional terms were listed

To investigate the biological functions and signal pathways of DEGs, we performed the GO annotation and KEGG pathway analysis. As shown in Fig. 3A–C, GO enrichment analysis demonstrated that DEGs were mainly enriched in some immunity-related processes (e.g., T cell activation, positive regulation of cytokine production, MHC protein complex, cytokine activity, CCR chemokine receptor binding). Moreover, analysis of the KEGG signal pathways revealed that DEGs were significantly annotated to inflammatory or immune-related processes (e.g., cytokine-cytokine receptor interaction, chemokine signaling pathway, and antigen processing and presentation) (Fig. 3D). The results of the enrichment analysis suggested that these inflammatory or immune-related processes may play an important role in the occurrence and progression of PBC.

### Identification of DEGs between high- and low-risk PBC patients

**Fig. 4:**
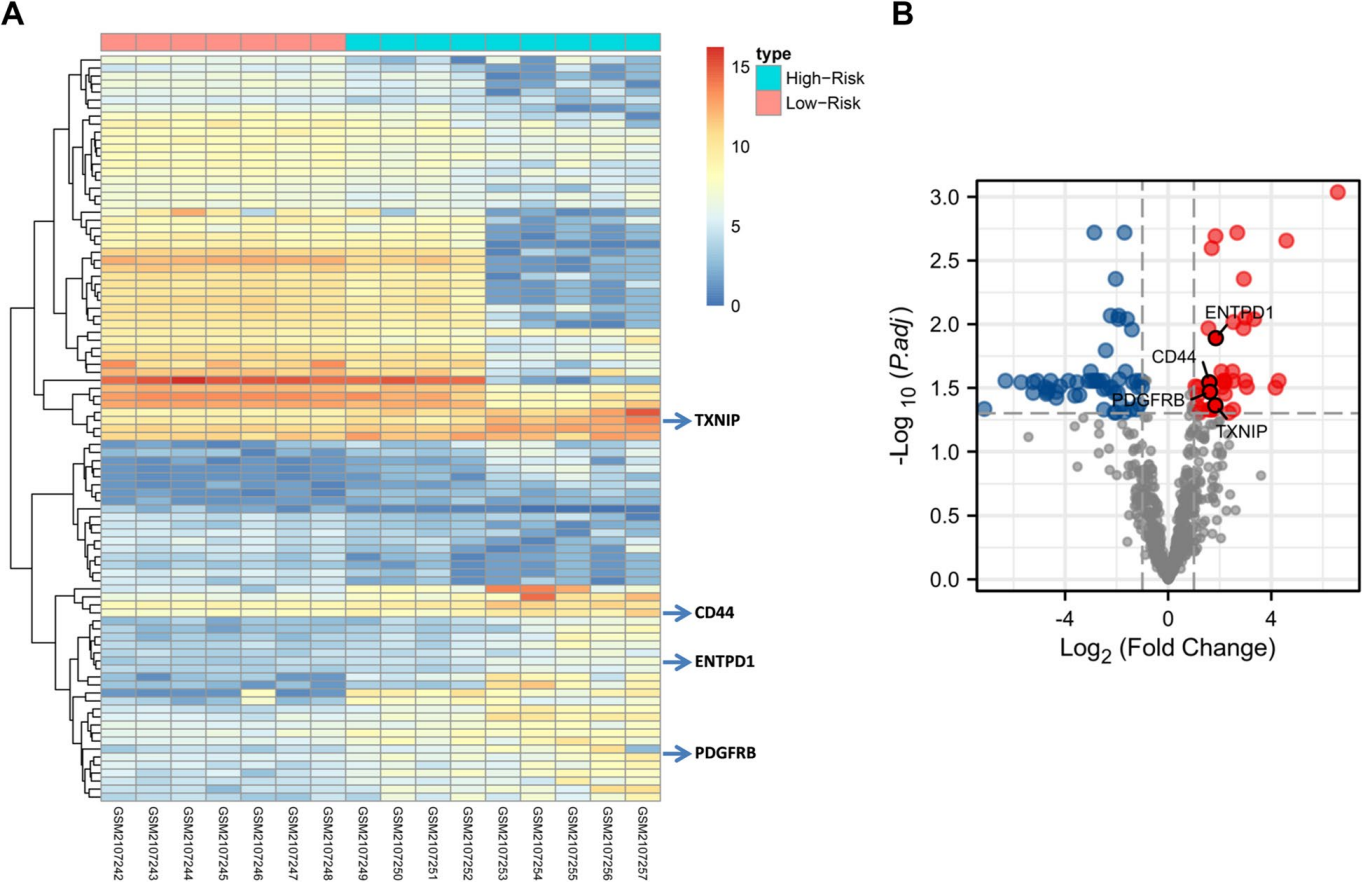
Identification of differentially expressed genes (DEGs) between the high- and low-risk PBC patients. **A** Heatmap analysis of all DEGs between high- and low-risk groups. **B** Volcano plots of DEGs, blue circles represented downregulated genes and red circles represented upregulated genes. (TXNIP: |Log2FC|= 1.832 *P*-adj = 0.043, ENTPD1: |Log2FC|= 1.848 *P*-adj = 0.013, CD44: |Log2FC|= 1.597 *P*-adj = 0.029, PDGFRB: |Log2FC|= 1.619 *P*-adj = 0.034)

In an attempt to further explore the heterogeneity of PBC patients, we carried out a subgroup analysis based on the risk of disease progression. From the analysis of differentially expressed DEGs between high- and low-risk patients, we obtained a total of 93 DEGs. The expression heatmap of DEGs is depicted in Fig. 4A. The distribution of DEGs was presented by the volcano plot, containing 39 upregulated genes and 54 downregulated genes (Fig. 4B). Simultaneously, function enrichment analysis of these DEGs was performed. It could be found that some immune-related processes, such as cytokine-cytokine receptor interaction and chemokine signaling pathway, were also enriched in the high-risk group (Supplementary Figure 1).

### Selection of hub genes

**Fig. 5:**
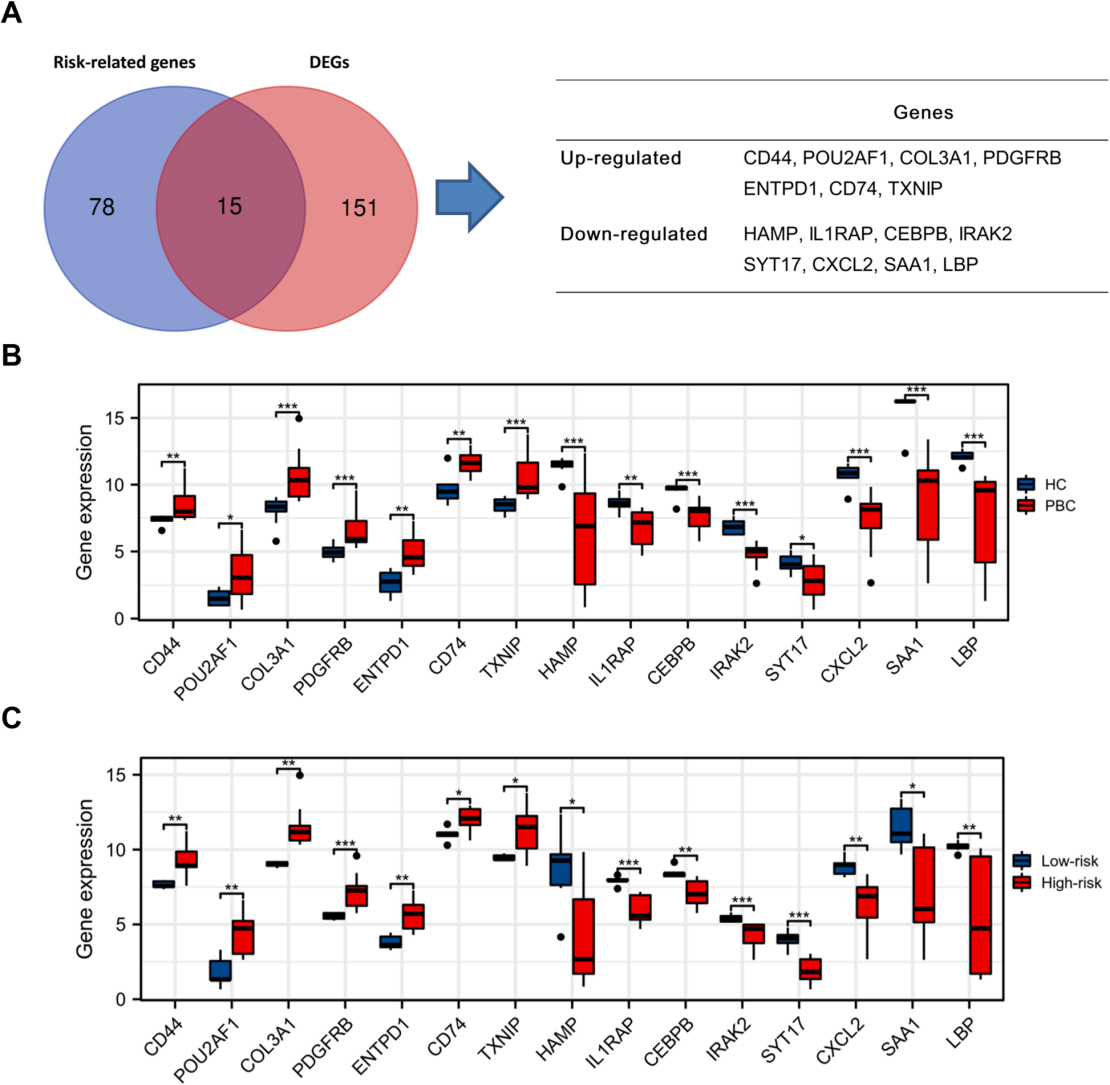
Exploration of differentially expressed genes (DEGs) for evaluation of disease progression. **A** Venn diagram showing the risk-related DEGs. **B** The expression patterns of 15 genes in health controls and PBC patients. **C** The expression patterns of 15 genes in high- and low-risk PBC patients. *P < 0.05, **P < 0.01, ***P < 0.001

**Fig. 6:**
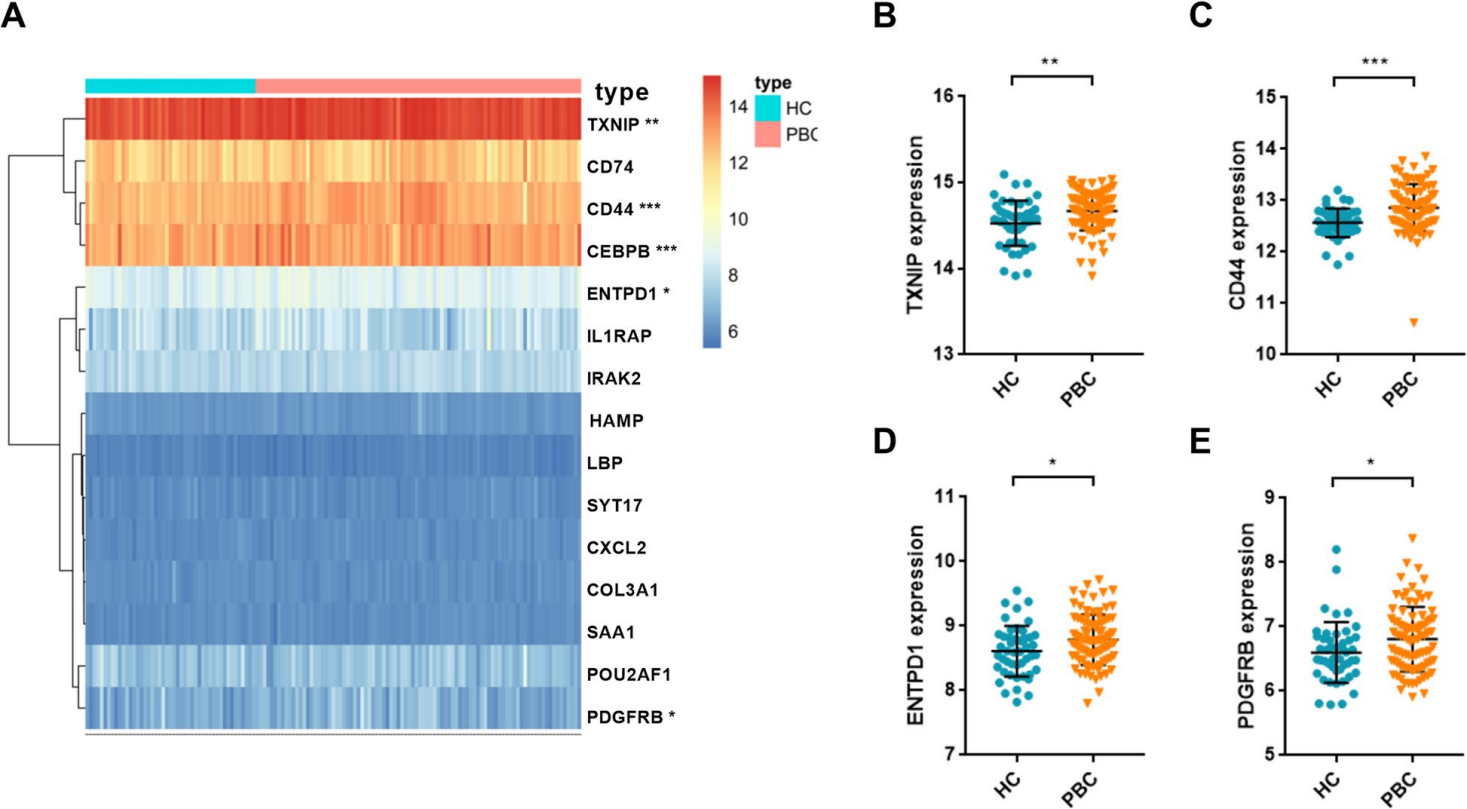
Validation of risk assessment markers in peripheral blood samples from cohort GSE119600. **A** Heatmap of 15 risk-related differentially expressed genes. The expression of TXNIP, CD44, ENTPD1, and PDGFRB was significantly upregulated in PBC samples compared with health controls. *P < 0.05, **P < 0.01, ***P < 0.001

Together with the above analysis, a total of 166 genes were differentially expressed between PBC samples and healthy controls, and 15 of them were associated with disease progression (Fig. 5A). The selected genes included 7 up-regulated genes and 8 down-regulated genes. The expression patterns of these genes are depicted in Fig. 5B, C. Meanwhile, we used ROC curves to evaluate the predictive power of 15 genes in the risk assessment of disease progression. The AUC value > 0.85 in all 15 genes indicated the high diagnostic value of these markers for high-risk PBC (Supplementary Figure 2). Next, considering the invasive nature of liver biopsy, we attempted to translate these tissue-based genes into a noninvasive clinical application. An independent public database, namely GSE119600, was used to substantiate the expression patterns of these indicators. Compared with healthy controls, five of these fifteen genes were statistically significant (Fig. 6A). Moreover, TXNIP, CD44, ENTPD1, and PDGFRB were consistent with the expression pattern detected in tissue samples (Fig. 6B–D). These genes were defined as core risk-related genes and used for subsequent analysis. Meanwhile, the above enrichment analysis suggested that CD44 was involved in the T cell activation and PDGFRB was involved in cytokine-cytokine receptor interaction. These biological processes are closely related to the inflammation and immune response, indicating these hub genes may participate in the occurrence and development of PBC.

### Validation of hub genes in animal models

**Fig. 7:**
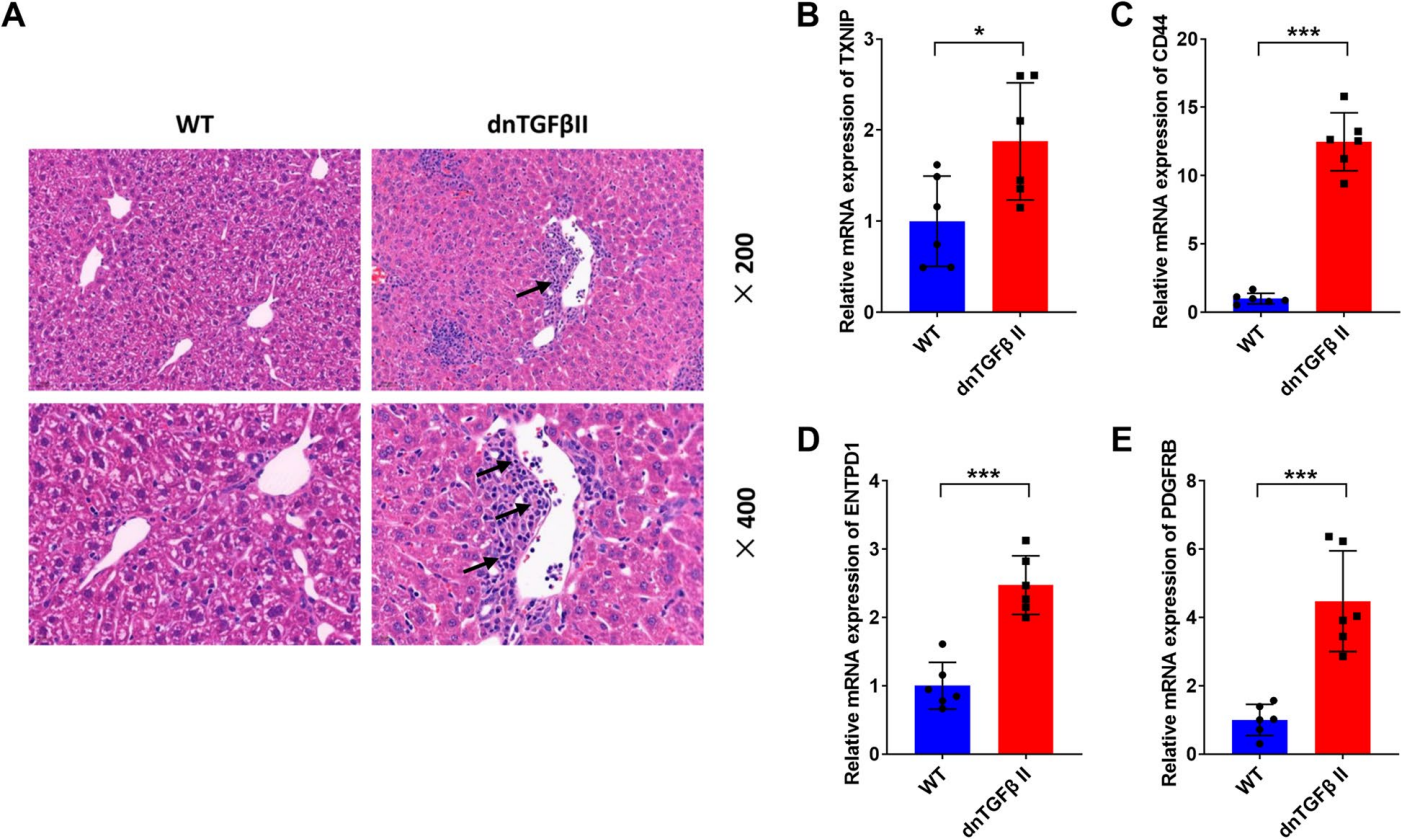
Verification of four hub genes in PBC model mice. **A** Histological features of the 12–14 weeks liver of wild types and dnTGF-β RII mice. The typical pathology was designated by the black arrow, characterized by a heavy infiltration of lymphocytes in the portal areas of the liver. **B**–**E** The expression of TXNIP, CD44, ENTPD1, and PDGFRB was significantly upregulated in dnTGF-β RII mice compared with wild-type mice. *P < 0.05, ***P < 0.001

The dominant-negative TGFβ receptor type II (dnTGF-β RII) mouse is a classical PBC animal model [32, 33]. The typical pathology is characterized by a heavy infiltration of lymphocytes in the portal areas of the liver, which is consistent with the liver pathological phenotype of PBC patients (Fig. 7A). In the present study, we examined the expression of core genes in the liver of 12–14-week-old dnTGF-β RII mice using the qRT-PCR. The results showed significantly elevated expression of these four core genes in dnTGF-β RII mice compared with normal controls (Fig. 7B–E). Besides, we examined the expression of these hub genes in the peripheral blood of dnTGF-β RII mice. As shown in Supplementary Figure 3, these genes displayed similar expression pattern.

### Development of a three-gene panel for screening high-risk PBC patients

**Fig. 8:**
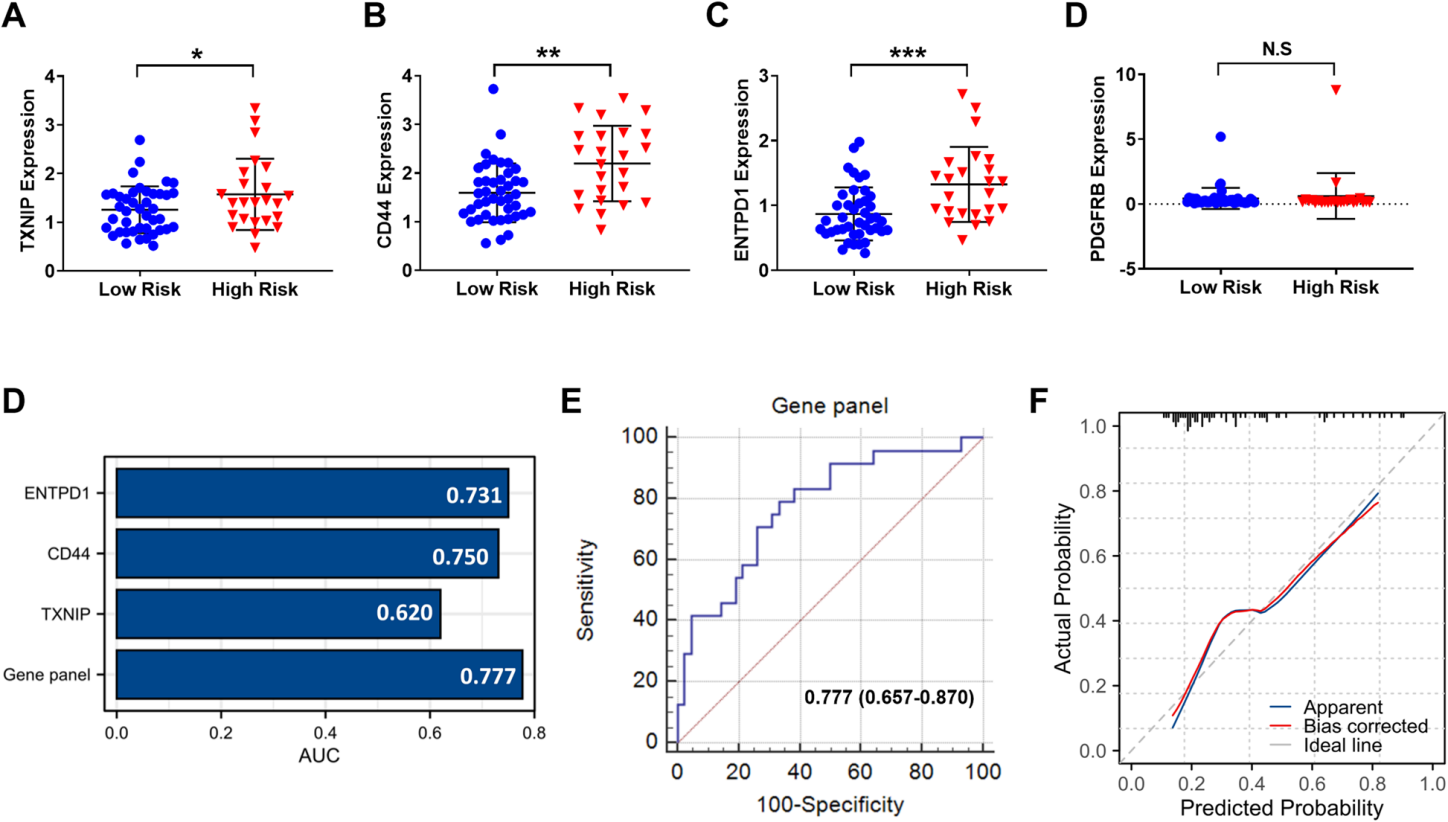
Examine the expression of four core genes in our validation cohort and develop a three-gene panel. The expression of **A** TXNIP, **B** CD44, **C** ENTPD1, and **D** PDGFRB in peripheral blood samples from 66 patients. **E** The area under the curve (AUC) of individual gene and the three-gene panel. **F** The ROC curve for the prediction of high-risk PBC patients according to the three-gene panel. **G**. The Calibration curve of the three-gene model. The calibration curve was close to 45°, indicating that the model had good predictive performance. NS, no significance, *P < 0.05, **P < 0.01, ***P < 0.001

To further reveal the clinical significance of the key genes in the PBC, we collected peripheral blood samples from 66 PBC patients in our center. We firstly compared the gene expression files of 4 core risk-related genes using the qRT-PCR. The results showed that three genes (TXNIP, CD44, and ENTPD1) were significantly upregulated in peripheral blood from high-risk PBC patients, while the expression of PDGFRB was not statistically different between the two groups (Fig. 8A–D). Moreover, we performed a correlation analysis between these hub genes and liver function indicators. As shown in Supplementary Figure 4, the expression of these hub genes was positively correlated with markers of liver injury and cholestasis including alanine aminotransferase, aspartate aminotransferase, and alkaline phosphatase. Next, we evaluated if each individual gene or the three-gene panel may assist in differentiating high-risk patients from total patients. The area under the curves of TXNIP, CD44, ENTPD1, and the three-gene panel were 0.620, 0.731, 0.750, and 0.777, respectively (Fig. 8E). The three-gene panel showed the best predictive power in identifying high-risk PBC patients in the serum validation cohort (Fig. 8F). Meanwhile, the calibration curve of the three-gene panel showed that the predicted values were consistent with the actual values (Fig. 8G).

Previous studies pointed out that ALP and TBiL were strong predictors of the prognosis of PBC patients, and they still have good predictive efficacy even in the analyses stratified by age, disease course, and drug therapy [34, 35]. To improve the performance of the three-gene panel, a refined model that integrated these indicators with the three-gene panel was constructed. The ROC curves showed that the combined model resulted in higher predictive accuracy (Supplementary Figure 5A-C). In addition, the calibration degree of the prediction model was also satisfactory (Supplementary Figure 5D-F).

## Discussion

Primary biliary cholangitis is a chronic progressive cholestatic liver disease that can progress to cirrhosis if lacking effective therapeutic interventions. The risk assessment for disease progression of PBC is usually based on a variety of biochemical criteria. However, these criteria have some limitations and cannot be used for early evaluation of therapeutic response. For example, some widely accepted standards like Barcelona, Paris-I, and Paris-II criteria often need 12 months to assess therapeutic effects. Zhang et al. tried to optimize the criteria and suggested to advance the time window for evaluation. They pointed out that biochemical response at the 6 months might be used in place of those evaluated after 1 year of UDCA therapy [36]. However, these criteria are just based on biochemical indicators. There is still a lack of biomarkers that can early identify patients who are at high risk of progression in clinical practice. Therefore, it is crucial to identify new and effective biomarkers facilitating risk stratification.

Ben Barron et al. showed that serum levels of CXCL11 and CCL20 could identify high-risk patients with a high degree of accuracy [37]. Besides, miR-125b, let-7b, and miR-520a-5p were suggested to be potential biomarkers for refractory PBC [38]. Ewa et al. validated that the activity of autotaxin is upregulated in refractory PBC patients and was related with poor survival [39, 40]. However, the definition of “high-risk” patients is fully dependent on the status of biochemical response. Our findings also showed that there were some differences among the various biochemical criteria (data are not shown). In fact, these biochemical criteria are just used as surrogate endpoints for long-term clinical outcomes. So, the application of hard endpoint, like liver transplantation, may enhance the reliability of the conclusion. In addition, the results of these studies were mostly inferred from serum; the expression level of these genes in the liver was unknown.

In the present work, we reanalyzed the GSE79850 and identified 15 candidate genes for the assessment of the risk of disease progression. In clinical settings, PBC is diagnosed when two of three criteria are met: elevated serum ALP, detection of AMA or other disease-specific autoantibodies, and typical histologic features by liver biopsy [6]. From this, liver biopsy is not indispensable for the diagnosis of PBC. So, examining these biomarkers in peripheral blood may be a better way to translate these tissue-based genes into a noninvasive routine clinical application. We used the GSE119600 to verify the result and identified 4 core genes (TXNIP, CD44, ENTPD1, and PDGFRB), which displayed similar expression pattern both in the liver and in peripheral blood. The expression level of four core genes in the liver or peripheral blood of PBC mouse model was also increased, implying that these genes may be involved in PBC pathogenesis. Finally, to confirm that the markers were repeatable for clinical use, we also used samples from PBC patients in our center to explore the predictive value of individual marker or the combined gene panel. Compared with low-risk patients (defined by the POISE criteria, ALP < 1.67 × ULN and bilirubin < 1 × ULN), the expression level of TXNIP, ENTPD1, and CD44 was upregulated in high-risk PBC patients with statistical significance. So, these three markers may be used in monitoring the disease progression of PBC patients. And the three-gene panel achieved a good predictive value with an AUC of 0.777 (95% CI: 0.657–0.870).

In addition to facilitating the risk assessment, identifying disease biomarkers may contribute to understanding disease mechanisms. In our study, functional enrichment analysis showed that these risk-related genes were mainly engaged in T cell activation, positive regulation of cytokine production, and cytokine-cytokine receptor interaction. It is well known that PBC patients have greater immune activation and higher cytokine levels than healthy people [41]. Enrichment analysis in our study also verified that signaling pathways regulating inflammation and the immune response were obviously enriched in the disease state.

Thioredoxin-interacting protein (TXNIP), also known as thioredoxin-binding protein 2 (TBP2), involves a reduction–oxidation (redox) signaling complex and has a pivotal role in mediating oxidative stress and inflammation in many diseases [42, 43]. Previous studies demonstrated that TXNIP could directly activate NOD-like receptor protein 3 (NLRP3) inflammasome, which regulated the expression of IL-1β and IL-18 [44]. Meanwhile, Mick et al. showed elevated IL-1β and NLRP3 activation correlated with disease activity in PBC patients [45]. In our study, our results also showed that the expression of TXNIP was upregulated in the liver and serum. Thus, reasonable hypothesis could be made that TXNIP might be a promising target in attenuating liver inflammation in PBC patients. ENTPD1 was the member of ectonucleoside triphosphate diphosphohydrolase family and expressed on the surface of innate and adaptive immune cell subsets, such as monocytes, dendritic cells, and T/B cells [46]. The function of the ENTPD1 was regarded as important modulators of the immune system, contributing to the balance between regulatory and effector lymphocytes in rheumatoid arthritis, Crohn’s disease, and autoimmune hepatitis [47]. The clinical values or biological roles of ENTPD1 have not been reported in PBC. Here, our results showed that the expression of ENTPD1 was significantly upregulated in PBC patients, especially in high-risk patients. CD44 is a major receptor for hyaluronic acid and has been extensively studied in tumors. Recently, studies on the role of CD44 in inflammation have been reported. Presumably, CD8^+^T cells expressing high levels of CD44 kill endothelial cells, resulting in massive extravasation of monocytes and CD4^+^T cells in the subarachnoid space [48]. Moreover, Qiang et al. demonstrated that CD44 deficiency resulted in reduced proinflammatory cytokine production in PCV2-induced lung of mice, and alleviating the pooling of T cells to the site of inflammation [49]. The liver pathology of PBC patients is characterized by the infiltration of a large number of lymphocytes in the portal area. All the above studies suggest that CD44 plays a role in promoting the recruitment of inflammatory cells, which is consistent with the elevated expression of CD44 found in high-risk PBC patients.

The merit of our study is that the three-gene panel was constructed based on multiple independent cohorts. Liver tissue samples are more helpful to reflect the pathology of the disease, while blood samples serve more for clinical translation. Admittedly, there were some limitations in our study. First, the sample size for analysis and verification is relatively small. Second, in the validation phase of gene panel, we just conducted a cross-sectional design. It is still necessary to gather more data to conduct a prospective cohort study. Third, the current research merely analyzed the microarray datasets of PBC at the transcription level, without the involvement of genomics, proteomics, and metabolomics. In the future, integrated multi-omics analysis will shed further light on the disease pathogenesis and mechanisms of disease progression.

## Conclusion

In summary, we identified three core genes (TXNIP, CD44, and ENTPD1) as potential biomarkers for the assessment of disease progression. Based on the above genes, we further developed a three-gene panel, which could help clinicians to early identify high-risk PBC patients to improve therapeutic strategies.

### Supplementary Information

**Additional file 1:** **Supplementary Figure 1.** Functional enrichment analysis of differentially expressed genes (DEGs) between the high- and low-risk PBC patients. (A) GO enrichment analysis contained three categories: biological process, molecular function, and cellular component. (B) KEGG pathway enrichment analysis. The top 10 functional terms were listed. **Supplementary Figure 2.** ROC curves of the 15 risk-related genes for the prediction of high-risk PBC patients in the GSE79850. **Supplementary Figure 3.** Validation of four hub genes in the peripheral blood samples of dnTGF-β RII mice. (A-D) Relative expression level of (TXNIP, CD44, ENTPD1 and PDGFRB) in wild types and dnTGF-β RII mice. ** *P* < 0.01, *** *P *< 0.001. **Supplementary Figure 4.** Correlation analysis between hub genes and liver functional indicators. **Supplementary Figure 5.** Predictive models integrated the gene panel with clinical parameters. (A-C) Receiver operating characteristic (ROC) curve analysis of the three models. (D-F) Calibration curve analysis of the three models. The calibration curve was close to 45°, indicating that the model had good predictive performance. **Supplementary Table 1.** Primers used in the Quantitative real-time PCR.

## Data Availability

The data in this study that support the findings are available in the GEO database (https://www.ncbi.nlm.nih.gov/geo/) with the following data accession number(s): GSE79850 and GSE119600.

## Abbreviations

ALP: Alkaline phosphatase
AMAs: Anti-mitochondrial antibodies
AUC: Area under the curve
DEGs: Differentially expressed genes
dnTGF-βRII: Dominant-negative TGFβ receptor type II
GEO: Gene Expression Omnibus
GO: Gene ontology
KEGG: Kyoto Encyclopedia of Genes and Genomes
LAMP-2: Lysosomal-associated membrane protein-2
MHC: Major histocompatibility complex
OCA: Obeticholic acid
PBC: Primary biliary cholangitis
RMA: Robust multiarray average
ROC: Receiver operating characteristic
TBiL: Total bilirubin
UDCA: Ursodeoxycholic acid
