# Supplementary material for: Integrative bioinformatics analysis and experimental validation of key biomarkers for risk stratification in primary biliary cholangitis
Source: Arthritis Res Ther. 2023 Oct 2;25:186. doi: 10.1186/s13075-023-03163-y (PMC10544390; doi:10.1186/s13075-023-03163-y)
Supplement: Supplementary file 1 — Additional file 1: Supplementary Figure 1. Functional enrichment analysis of differentially expressed genes (DEGs) between the high- and low-risk PBC patients. (A) GO enrichment analysis contained three categories: biological process, molecular function, and cellular component. (B) KEGG pathway enrichment analysis. The top 10 functional terms were listed. Supplementary Figure 2. ROC curves of the 15 risk-related genes for the prediction of high-risk PBC patients in the GSE79850. Supplementary Figure 3. Validation of four hub genes in the peripheral blood samples of dnTGF-β RII mice. (A-D) Relative expression level of (TXNIP, CD44, ENTPD1 and PDGFRB) in wild types and dnTGF-β RII mice. ** P < 0.01, *** P < 0.001. Supplementary Figure 4. Correlation analysis between hub genes and liver functional indicators. Supplementary Figure 5. Predictive models integrated the gene panel with clinical parameters. (A-C) Receiver operating characteristic (ROC) curve analysis of the three models. (D-F) Calibration curve analysis of the three models. The calibration curve was close to 45°, indicating that the model had good predictive performance. Supplementary Table 1. Primers used in the Quantitative real-time PCR. [file 13075_2023_3163_MOESM1_ESM.docx]

**Supplementary Figure 1. Functional enrichment analysis of differentially expressed genes (DEGs) between the high- and low-risk PBC patients.** (A) GO enrichment analysis contained three categories: biological process, molecular function, and cellular component. (B) KEGG pathway enrichment analysis. The top 10 functional terms were listed.


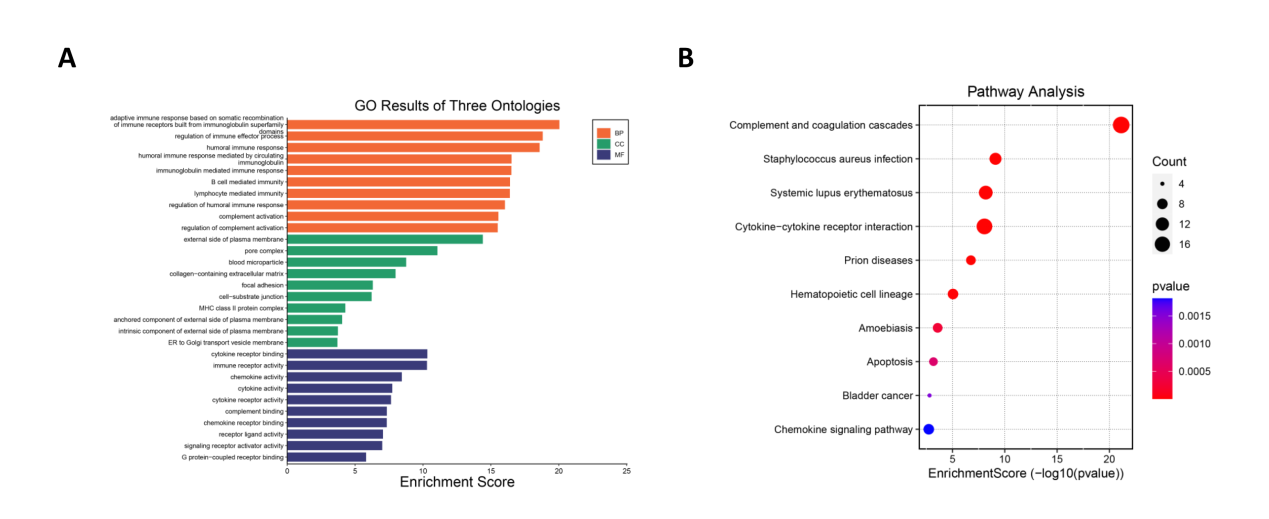


**Supplementary Figure 2. ROC curves of the 15 risk-related genes for the prediction of high-risk PBC patients in the GSE79850.**


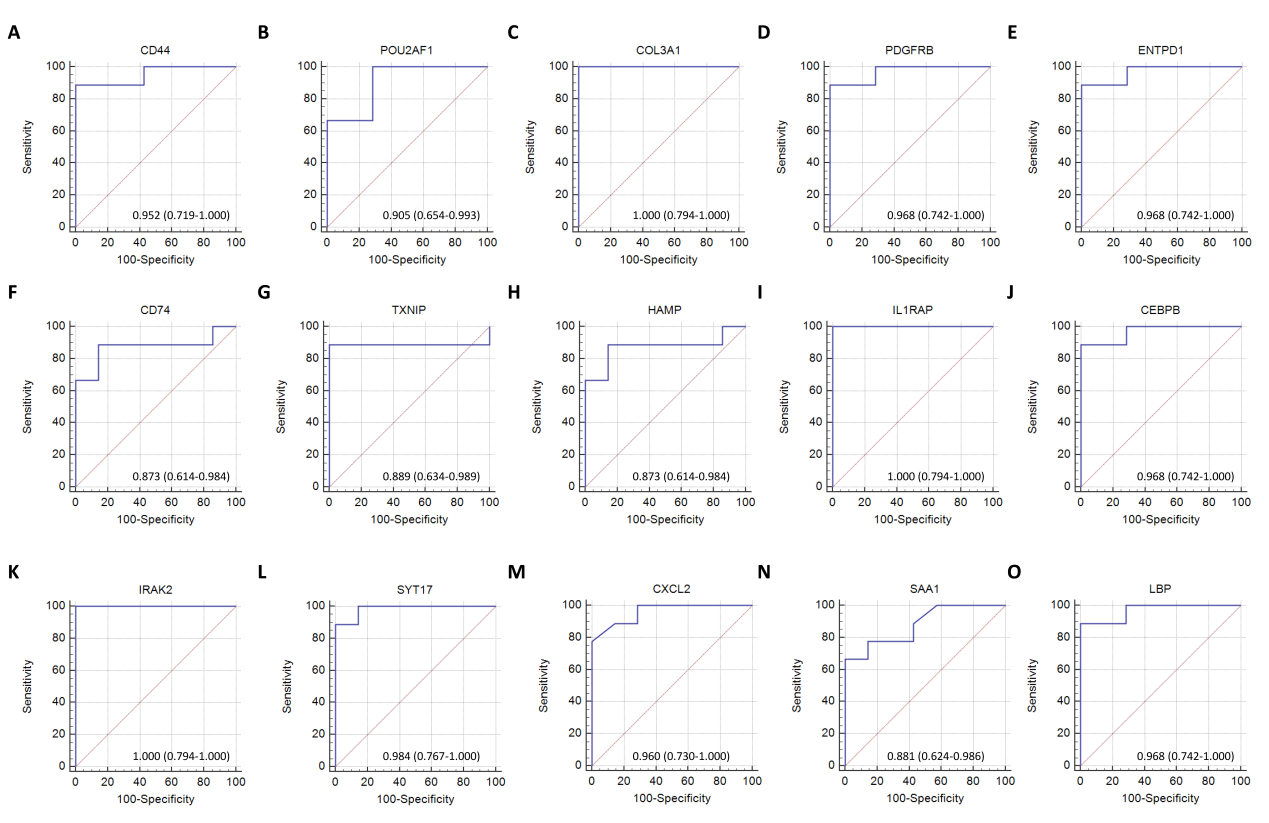


**Supplementary Figure 3. Validation of four hub genes in the peripheral blood samples of dnTGF-β RII mice.** (A-D) Relative expression level of (TXNIP, CD44, ENTPD1 and PDGFRB) in wild types and dnTGF-β RII mice. ** *P* < 0.01, *** *P* < 0.001.


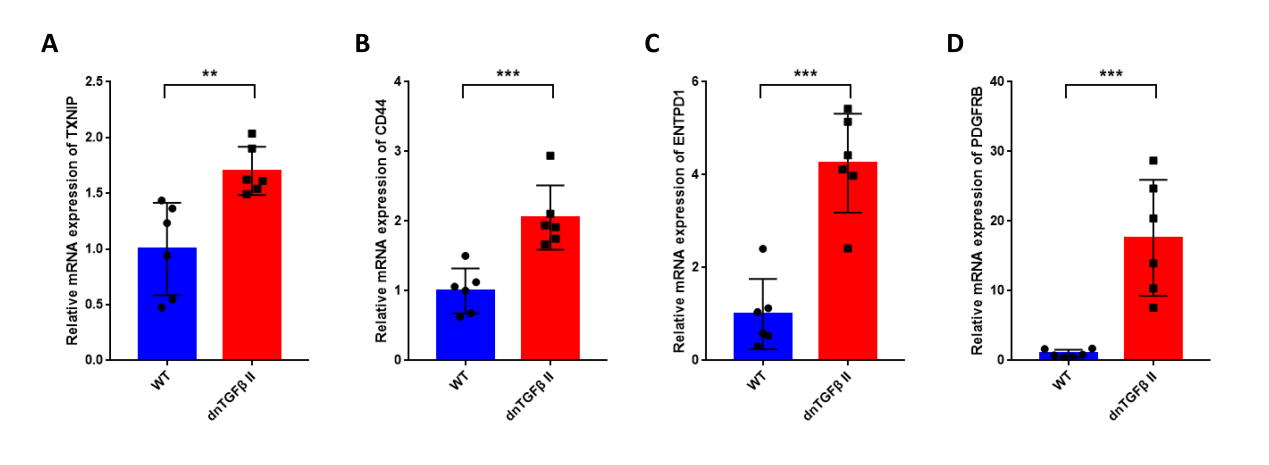


**Supplementary Figure 4. Correlation analysis between hub genes and liver functional indicators.**

**
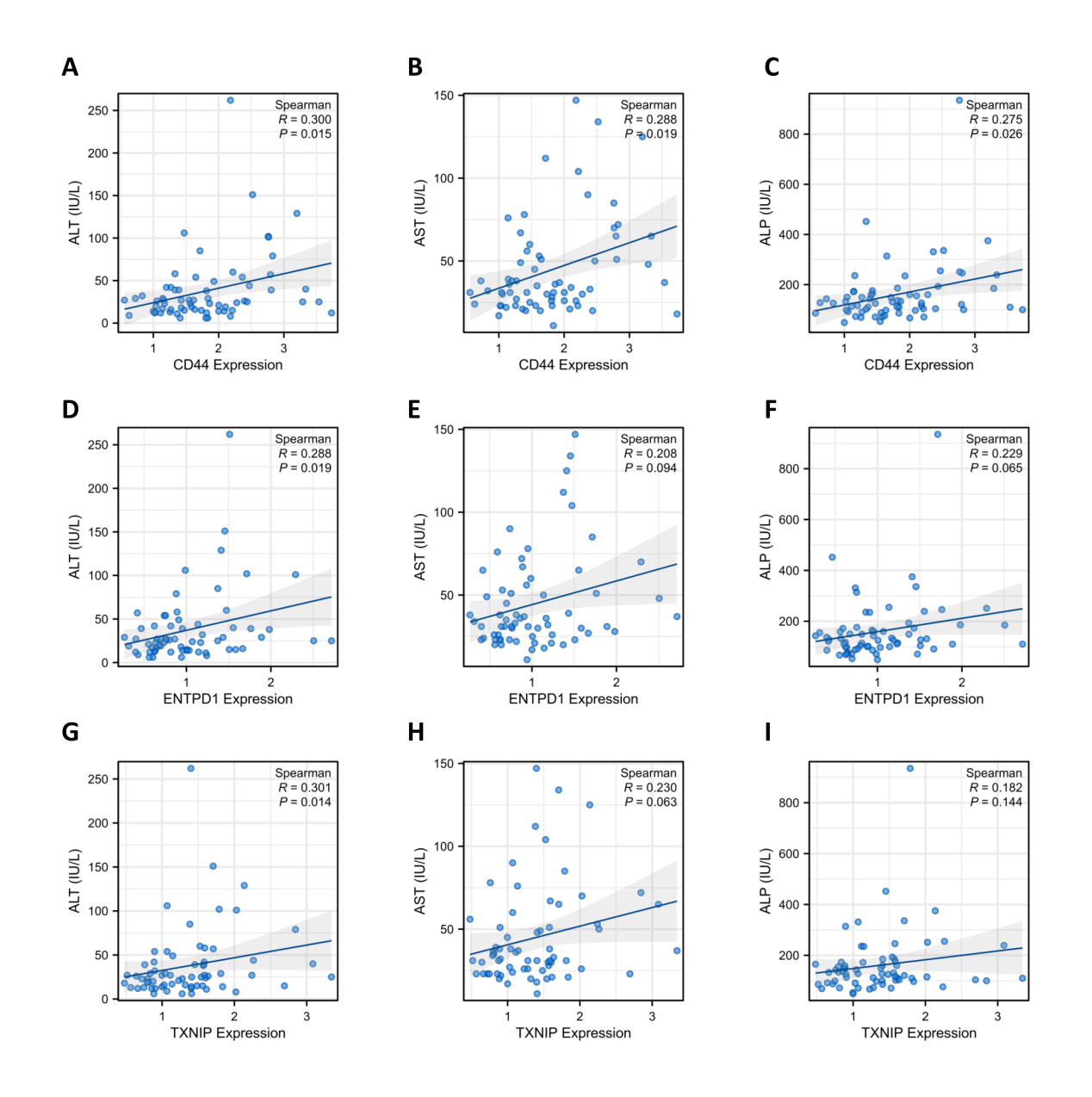
**

**Supplementary Figure 5. Predictive models integrated the gene panel with clinical parameters.** (A-C) Receiver operating characteristic (ROC) curve analysis of the three models. (D-F) Calibration curve analysis of the three models. The calibration curve was close to 45°, indicating that the model had good predictive performance.

**
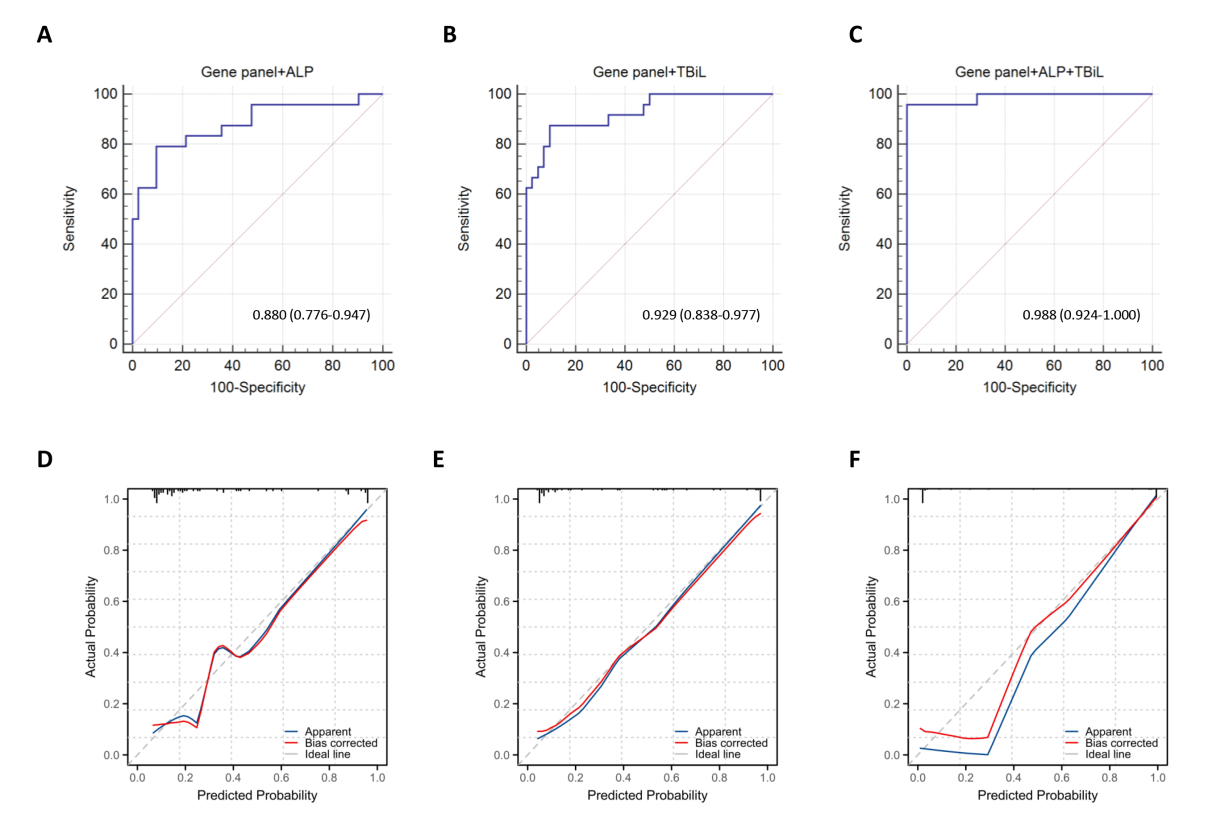
**

**Supplementary Table 1. Primers used in the Quantitative real-time PCR.**

| **Gene** | **Forward Primer** | **Reverse Primer** |
| --- | --- | --- |
| **β-actin (mmu)** | CAGCACAATGAAGATCAAGATC | CGGACTCATCGTACTCCTGCTT |
| **CD44 (mmu)** | TCGATTTGAATGTAACCTGCCG | CAGTCCGGGAGATACTGTAGC |
| **TXNIP (mmu)** | GGCCGGACGGGTAATAGTG | AGCGCAAGTAGTCCAAAGTCT |
| **ENTPD1(mmu)** | AAGGTGAAGAGATTTTGCTCCAA | GCATCCAACACAATCCCATACT |
| **PDGFRB (mmu)** | AGGAGTGATACCAGCTTTAGTCC | CCGAGCAGGTCAGAACAAAGG |
| **β-actin (hsa)** | CTCCATCCTGGCCTCGCTGT | GCTGTCACCTTCACCGTTCC |
| **CD44 (hsa)** | CTGCCGCTTTGCAGGTGTA | CTGCCGCTTTGCAGGTGTA |
| **TXNIP (hsa)** | CTCGTGTCAAAGCCGTTAGGA | TCTCATTCTCACCTGTTGGC |
| **ENTPD1 (hsa)** | AGGTGCCTATGGCTGGATTAC | CCAAAGCTCCAAAGGTTTCCT |
| **PDGFRB (hsa)** | AGCACCTTCGTTCTGACCTG | TATTCTCCCGTGTCTAGCCCA |
